# The Current State of Weight-Cutting in Combat Sports

**DOI:** 10.3390/sports7050123

**Published:** 2019-05-21

**Authors:** Oliver R. Barley, Dale W. Chapman, Chris R. Abbiss

**Affiliations:** 1 Centre for Exercise and Sports Science Research, School of Medical and Health Sciences, Edith Cowan University, Joondalup, WA 6027, Australia; c.abbiss@ecu.edu.au; 2 Performance Support—Physiology and Nutrition, New South Wales Institute of Sport, Sydney Olympic Park, NSW 2127, Australia; dale.chapman@NSWIS.com.au

**Keywords:** Combat sports, weight loss, martial arts, weight cutting, hydration, thermoregulation

## Abstract

In combat sports, athletes are divided into categories based on gender and body mass. Athletes attempt to compete against a lighter opponent by losing body mass prior to being weighed (i.e., ‘weight-cutting’). The purpose of this narrative review was to explore the current body of literature on weight-cutting and outline gaps for further research. Methods of weight-loss include energy intake restriction, total body fluid reduction and pseudo extreme/abusive medical practice (e.g., diuretics). The influence of weight-cutting on performance is unclear, with studies suggesting a negative or no effect. However, larger weight-cuts (~5% of body mass in <24 h) do impair repeat-effort performance. It is unclear if the benefit from competing against a smaller opponent outweighs the observed reduction in physical capacity. Many mechanisms have been proposed for the observed reductions in performance, ranging from reduced glycogen availability to increased perceptions of fatigue. Athletes undertaking weight-cutting may be able to utilise strategies around glycogen, total body water and electrolyte replenishment to prepare for competition. Despite substantial discussion on managing weight-cutting in combat sports, no clear solution has been offered. Given the prevalence of weight-cutting, it is important to develop a deeper understanding of such practices so appropriate advice can be given.

## 1. Introduction

The term ‘combat sports’ refer to a class of contact sports where competitors engage in one-on-one combat with the specific ruleset of the bout depending on the specific combat sport. Such sports can involve striking techniques such as punches and kicks, grappling techniques such as chokes and joint-locks, or a mixture of both striking and grappling techniques [1]. Combat sports are typically divided into grappling, striking or mixed-style disciplines. Grappling sports focus on the grappling component of combat to the exclusion of striking techniques, whereas striking sports do the inverse. Mixed-style sports include a mixture of both grappling and striking but sometimes still focus on one aspect more than the other [1]. In an effort to ensure competitors of a similar size, competition organisers divide competitors by their body weight [2]. However, it is common practice in combat sports for athletes to attempt to gain a competitive edge and be paired with a smaller opponent by losing significant body weight in the days and weeks leading up to being weighed [3,4,5,6]. This practice has been colloquially referred to as ‘weight-cutting’ and appears to be present at least to some degree in every combat sport, though the magnitude ranges between different disciplines [3,4,5,6]. 

There has been a substantial but limited body of scientific research investigating the prevalence of weight-cutting in different combat sports [3,4,5,6], the different methods of weight loss [3,4,5,6,7,8], the influence on competitive performance [9,10,11,12], the physiological changes associated with weight-cutting [9,13,14,15,16,17] and methods to improve performance following weight-cutting [2,18]. Within this body of literature, there are conflicting findings on many of the topics mentioned above. For example, studies have indicated that weight-cutting has a negative effect on exercise performance [9,10,13], while other studies have reported no effect [11,19,20]. The limited volume of literature across a range of topics within weight-cutting precludes a comprehensive systematic review which would use a highly technical, specific methodological approach to identify and appraise evidence on weight-loss practices in different combat sports [21]. Contrastingly, the purpose and direction employed forthwith is that of a narrative review, which seeks to provide clarification and nuanced insight into weight-cutting in different combat sports with an interpretive and discursive synthesis of the existing literature. Consequently, this narrative appraisal of the literature allows for an interpretive overview, providing reflection and context rather than a formal objective appraisal of a weight-cutting practices in the constraints of rigidly defined inclusion criteria. This aspect of narrative reviews should be viewed as a strength rather than a weakness in that it allows for nuanced commentary on a topic that can provide valuable information for future research [21]. Therefore, the purpose of this review is to critically evaluate the current body of research on weight-cutting in combat sports, including prevalence, common practices, performance effects, physiological response, and potential strategies to maximise performance following weight-cutting to provide clarity on this convoluted topic.

## 2. Prevalence, Magnitude and Methods of Weight-Cutting

Before considering the prevalence, magnitude and different methods of weight-cutting, it is important to consider the different rules and regulations across the combat sports. The time between weigh-in and the competition differs between sports, which would plausibly influence the weight loss strategies employed by athletes [2,22]. The time between weigh-in and competition ranges from 2 up to 24 h depending on the sport and level of competition. Regardless, between 60–80% of competitive combat sports athletes have reported to engage in some form of weight-cutting, including athletes from combat sports such as mixed martial arts (MMA), Brazilian jiu jitsu (BJJ), jujitsu, taekwondo (TKD), boxing, judo, Muay Thai/kickboxing and wrestling [3,6,23,24]. The weight loss practices are not identical between combat sports, with the prevalence of weight-cutting being greater in boxing and MMA [3,23]. While the current body of research does appear to be consistent, it is important to note that the majority of data was collected via self-reported methods. Self-reported data has been observed to be less accurate than direct observation due to subject measurement bias [25]. Future research would benefit from collecting unbiased observational data of athletes during weight-cuts and making statistical comparisons between combat sports in an attempt to replicate the prevalence observed in the current data.

A plethora of weight loss methods have been utilised prior to competition across combat sports including energy intake restriction (gradual dieting and fasting); total body fluid reduction (restricting fluid intake, increasing sweat response (heated wrestling, plastic suits, saunas and spitting) and; pseudo extreme/abusive medical practice (laxatives, diet pills, diuretics, enemas, sporting bulimia (vomiting)) [5,26]. It appears that gradual dieting and increased energy expenditure through exercise are common among all combat sports that have been investigated [3,5,6,24]. However, the magnitude of the increase in the volume of exercise is not clear, nor the type of exercise utilised. This is important because drastic changes in training volume along with a reduction of energy consumed could increase the risk of overreaching/overtraining leading up to competition if not properly planned [27]. While more extreme methods of energy intake manipulation such as skipping meals and fasting are less common, they still appear to at least be occasionally used by a large portion of combat sports athletes [3,6]. Unfortunately, there is minimal research investigating the specific dietary changes athletes implement at different points of their weight loss protocols. Though self-reported data has shown that athletes utilise carbohydrate restriction more commonly than fat restriction; however, specifics of the diet were not investigated [24]. These trends could be a result of the recent increase in popularity of low-carbohydrate high-fat diets within athletic populations [28]. Clearly further research is needed to investigate the efficacy of different diets when attempting to make weight for combat sports. 

In short time periods (<24 h), the majority of body mass reduction will likely come from body fluid manipulation. Indeed, 45–75% of the average human’s body mass consists of water [29,30] and as such, manipulation of body fluid can result in rapid acute losses of body mass [9]. Both a restriction of fluid ingestion and an increase in thermal strain to induce sweating appear to be commonplace in a wide range of combat sports [3,6,24], with losses of over 5% of athlete body mass reported within 24 h of the weigh-in [6,23]. The most common methods of inducing thermal strain for weight loss in combat athletes appear to be the use of a sauna (or heated rooms) and the use of sweatsuits [3,24]. However, only a narrow range of methods to induce thermal strain have been included in surveys [3,5,6]. It is therefore possible that athletes utilise other methods (such as towel wrapping) that have not been systematically investigated and reported in scientific literature. As different methods of inducing thermal strain may have differing effects on an individual’s physiology [31] and possibly performance, investigating other potential methods that athletes use to induce thermal strain is important. Finally, there is also evidence that many combat sports athletes are engaging in a practice called ‘water-loading’ where athletes reduce sodium and overdrink water in an effort to trigger a ‘flushing mode’ where excessive urine production can be used to maximise fluid driven weight loss [3,23], but the use of this practice appears more commonplace in MMA than more traditional combat sports (such as boxing, judo, taekwondo and wrestling) [3,6,23]. There is research to suggest that water-loading is a safe and effective method of manipulating body mass (~3%) without impairing physical performance in grappling athletes [8]. However, more research is required to assess the viability of water-loading as a weight loss strategy in striking and mixed-style combat sports athletes and when used for larger (>4%) body mass reduction. The results of further research in this area could also apply to other sports that employ weigh-ins, such as rowing or body-building.

Other, less conventional methods such as the use of laxatives, diet pills, diuretics, enemas, sporting bulimia (vomiting) have also been used for weight loss in combat sports [3,5,6,24]. These practices do not appear as prevalent as fluid restriction and thermal strain [3,5], in fact, most athletes report never or almost never using such methods [3,4,5]. Regardless of the method of weight-cutting utilized, it is unclear if there are differences between different competitive levels (such as amateur compared to professional), which should be the topic of future research. 

## 3. Weight-Cutting and Performance

As weight-cutting is done in preparation for competition it is essential to understand the influence on performance. Studies correlating weight manipulation strategies to competitive outcomes in boxing have reported greater weight loss and regain to improve [32,33,34] or have no influence on competitive success [35,36]. The results of laboratory-based trials investigating the effects of weight-cutting on varying aspects of exercise performance are also mixed. Indeed, there is research suggesting that weight-cutting practices negatively influence repeat-effort performance [9,10,13,17,37], while other research indicates no impact on repeat-effort [11,19,20], aerobic [14], and anaerobic performance [38]. It appears that when larger magnitudes (>3% body mass) are lost rapidly (<5 h) using thermal strain, high-intensity exercise performance is impaired [9,13,17]. However, when a similar magnitude of weight is lost over multiple days (2–5) using a combination of methods (food restriction and body-fluid manipulation) laboratory-based trials have failed to observe any negative effect [11,19,20,38]. Studies that report a negative effect of weight-cutting on performance typically observe these effects on high-intensity repeat-effort performance, even up to 24 h following weight loss [9,13,17]. However, the relationship between weight-cutting and strength is far less clear [9,13,17]. As such, combat sports with a longer total competition duration are likely to be at greater risk of impaired performance resulting from weight-cutting practices. 

The larger magnitudes of body mass loss (>3%) that has been associated with performance decrements may not be relevant to traditional combat sports such as taekwondo, judo, jujitsu and karate as the magnitude of weight loss has been observed to be smaller than other combat sports such as MMA [6,23,24]. The magnitude of body mass loss in MMA has been suggested to be larger than other combat sports [6,23], so it seems possible that the weight-cutting in MMA will have a greater influence on competitive performance. However, it has been suggested that the competitive advantages yielded by weight-cutting may outweigh the potential negative affect on physical performance [39]. It is also unclear if there are any differences in weight loss magnitude or its effects on physical performance between different competitive levels, so if higher levels of competition lose more weight, it is possible that they would suffer greater detriments to performance resulting from such weight loss. Alternatively, it is possible that athletes at a higher level of competition would be better at manipulating their body mass and hence not be subject to the same declines in performance that lower level athletes are. Further research will be required to determine the influence of competitive level on performance following acute body mass manipulation. 

## 4. The Mechanisms by which Weight-Cutting Influences Performance

Given the varying weight loss methods used by athletes there are a wide range of potential physiological mechanistic pathways by which weight-cutting may influence performance. Acute reductions in energy intake are likely to influence performance through reduced glycogen concentration [7]. Lower levels of muscle glycogen have been shown to induce fatigue by impairing excitation–contraction coupling in the muscle cells [40]. Longer periods of energy restriction may influence the physiology of carbohydrate and lipid metabolism which could also affect exercise ability [41,42]. Indeed, prolonged periods of low carbohydrate intake will increase the body’s reliance on ketones as an energy source and potentially downregulate enzymes associated with anaerobic glycolysis [41,42], which could impair high-intensity exercise performance [28]. However, whether an increased reliance on ketones impairs high-intensity performance is still controversial [43,44]. Though it is important to note that there is a lack of research investigating whether the energy restricting strategies employed by combat sport athletes result in them competing in a state of ketosis which should be investigated by future research. Conversely, in the case of using acute dehydration as a weight loss method, different physiological changes are likely to influence exercise performance. Dehydration induced via sweat loss is commonly associated with a reduction in the blood plasma and thus total blood volume which would impair cardiovascular function, muscle blood flow and thermoregulatory capacity [45,46]. However, given the recovery period between weigh-in and competition can be as long as 24 h [9], it is unclear if a reduction in blood plasma will remain. Indeed, research investigating the recovery of body water following weight-cutting has found 24 h to be insufficient to recover hydration status [47] while other research has observed many markers of hydration to return to baseline following a 24 h recovery [9]. The mixed results may be due to the difficulty of assessing human hydration, especially following acute dehydration and rehydration [9], [48]. The potential changes in plasma volume may also combine with the observed decrease in haemoglobin mass following weight-cutting to impair aerobic ability [14,16]. However, previous research has observed that despite a decrease in haemoglobin mass, weight-cutting was not found to influence aerobic performance [14]. However, this study did not investigate other markers of aerobic fitness such as metabolic thresholds, economy and oxygen kinetics which may have been affected and would be an interesting avenue for future research. 

Acute dehydration may significantly alter electrolyte concentration which may influence the cell’s fluid balance, its metabolic processes and as a result impair neuromuscular function [45,49]. Indeed, electrolytes are essential for the maintenance of the membrane electrochemical potential and actin-myosin function [49]. However, research investigating the effects of dehydration on membrane electrochemical potential has been mixed with studies observing a negative [50,51,52,53], or no impact [54]. There is much less research investigating the influence of acute dehydration and rehydration on neuromuscular function. Acute dehydration has been observed to not influence maximal voluntary strength or central and peripheral neuromuscular function despite a negative effect on repeat-effort endurance [17]. Conversely, there is research observing changes in muscle electromyography to accompany a decline in endurance [50]. Future research should investigate the influence of acute dehydration using more comprehensive markers of central and peripheral neuromuscular function (using methods such as transcranial magnetic stimulation) to best determine what mechanisms may influence performance. Alternatively, it is possible that acute dehydration influences mental fatigue as previous research has observed increases in perception of exertion during exercise as well and negatively influencing mood-state [9,10,17]. It is important to note that the potential changes in physiology and psychobiology associated with acute dehydration and rehydration has not been comprehensively explored and requires further investigation. 

## 5. The Negative Health Implications of Weight-Cutting

Significant weight-cutting may not only compromise competitive performance, but may also be a risk to athlete wellbeing [39]. Severe (or even moderate) dehydration used for weight loss in weight restricted sports increases the risk of acute cardiovascular problems [39]. Indeed, the increased blood viscosity associated with dehydration would increase the risk of ischaemic heart disease and stroke [39,55]. It has also been proposed that significant levels of dehydration could also alter the brain morphology and potentially increase the risk of brain injury arising from head trauma induced by strikes [23,56]. This is due to decreased cushioning forces during head impacts resulting from changes in brain morphology associated with dehydration [23,56,57]. Additionally, the thermal exposure typically used to induce such dehydration puts athletes at an increased risk of heat illness such as heat stroke [39]. However, while there have been several athlete deaths when undergoing weight-cutting, there is a lack of research investigating the specific instance of cardiovascular complications and/or heat illness in combat sports which should be the topic of future research [7,23]. There are also other potential risks associated with weight-cutting, including hormonal imbalances, changes to insulin sensitivity, bone loss and suppressed immune function [39,58]. In fact, previous research has found weight-cutting to influence testosterone, growth hormone, sex hormone-binding globulin, growth-hormone binding protein, cortisol, insulin [59,60,61]. Such changes have been proposed to potentially influence bone mineral density, adolescent development and blood glucose regulation [59,60,61]. There has been some research to show that weight-cutting increases the risk of injury in judo athletes [62], but further research is needed to explore the incidence of illness and injury occurring acutely due to weight-cutting as well as over an athlete’s career in the long-term.

Weight-cutting does not only pose physical but also mental stress. Indeed, the process of going through a weight-cut is an exhausting combination of starvation, and heat exposure which imposes significant mental strain [17,39,63]. It is also unclear if the constant attention directed towards an athlete’s body mass increases the risk of developing an unhealthy body image and thus increasing the risk of eating disorders [64,65]. However, the increased prevalence of eating disorders has been well documented in other sports with a significant emphasis on body image such as gymnastics [66] and bodybuilding [67]. Despite the wide range of potential physical and psychological health risks associated with weight-cutting, there is alarmingly little research on such topics. With weight-cutting practices being prevalent in not only adults but also adolescents at all levels of competition [39], it is imperative that the potential physical and psychological effects are comprehensively explored by future research. 

There remains an unknown impact of weight-cutting practices on the overreaching and overtraining phenomenon. The identified health risk factors above are all known to have implications in the process of athletes developing overreaching [68] and thus it is unclear if weight-cutting places combat sports athletes at a greater risk of progressing into overtraining. It is a difficult scenario to determine the true implications of one factor on the other, importantly because pushing athletes truly into the overreaching is fraught with issues [69,70] and thus there is a need for work to consider how and where weight-cutting practices best fit into a periodised plan. For example, combat athletes do use contemporary tapering strategies [71] but it is unclear how additive psychological stressors of cutting weight impact the tapering process. 

## 6. The Period between Weigh-in and Competition

In most weight restricted sports, a recovery period is allowed between the weigh-in and the competition. It is recognised that following weigh-in it is essential for athletes to replenish carbohydrates, electrolytes and total body fluid to recover for competition. In some sports athletes are weighed the same day as competition, which results in 2 to 12 hours between the weigh-in and competition, whereas other sports weigh athletes the day before which allows longer time periods up to and beyond 24 h [2,9]. The time between the weigh-in and the competition would influence both the strategy of weight loss and recovery [7]. 

Carbohydrate utilisation is important in the repeat high-intensity efforts of most combat sports [7,72], especially in nonketogenic-adapted athletes. The replenishment of depleted carbohydrate stores has been extensively studied across a wide range of sports [7,72,73]. This research has addressed subjects such as the type of carbohydrates, the timing of ingestion, the amount of carbohydrates and other macronutrients to maximise glucose absorption [73,74]. Within combat sports specifically, carbohydrate intake of 5–10 g/kg/day has been recommended when recovering from weight-cutting as it should be sufficient to replenish the lost glycogen as well as improving the recovery of body mass [7]. Indeed, previous research has found that high carbohydrate refeeding improves exercise performance five hours following rapid body mass loss in combat sports athletes [75], conversely other research has found carbohydrate refeeding to not help following short (one hour) recovery periods [76]. However, it is important to consider athlete comfort as excessive carbohydrate consumption in short recovery periods may result in significant gastrointestinal distress which may influence competitive performance [7]. To maximise the nutrients that reach the bloodstream and minimise the occurrence of gastric discomfort, athletes will need to consider the gastric emptying rate. Higher energy density of the flood/fluid ingested and a smaller total volume in the stomach both slow down gastric emptying rate [77]. During shorter recovery periods, athletes will likely benefit from liquid carbohydrates to maximise gastric emptying rate [7,74,78]. Conversely, during longer recovery periods, athletes will be able to ingest more energy dense food/fluids and perhaps accompany the ingestion of carbohydrates with protein to increase glycogen synthesis [7]. However, there is a paucity of research investigating different refeeding strategies following weight-cutting, especially regarding the longer recovery periods allowed in sports like MMA.

In addition to the recovery of muscle glycogen, athletes need to consider their total body water and electrolyte balance. Gastric emptying rate will also influence the replenishment of total body water as the majority of fluid intake enters the bloodstream from the small intestine [77]. As a result, the ingestion of fluid and electrolytes must be managed similarly to carbohydrate intake by taking into account the duration of the recovery period [7,77]. Different to carbohydrates, there is no mechanism for active transport of water from the gut so the osmolality of the fluid ingested will have a large impact on absorption [77]. The concentration of electrolytes in the body will influence how much fluid is retained in the blood and ultimately the cells [7,77]. Excessive consumption of hypotonic fluids following dehydration will result in excessive water being excreted before the intracellular and extracellular fluids equilibrate, thus reducing the efficiency of rehydration [7,79]. It is important to note that the consumption of electrolytes is essential when dehydration is induced using sweat loss but not as important when induced exclusively through fluid restriction as less electrolytes are lost [7]. During the recovery period, athletes should endeavour to ingest 125–150% of the fluid lost, but this may not always be realistic (especially when only a short recovery period is allowed) as it can induce gastrointestinal discomfort which would likely be a greater detriment to performance than the dehydration [7]. However, more research is needed to investigate the efficacy of specific rehydration strategies following weight-cuts in combat sports athletes.

## 7. Strategies for Managing Weight-Cutting in Combat Sports

Given that weight-cutting has serious health risks and may negatively influence exercise performance, it is not surprising that conversations both inside and out of the scientific community have been had around managing the practice of weight-cutting [23,39,64]. This has included suggestions of policies to minimise weight-cutting [23,64] and calls to ban weight-cutting altogether [39]. Suggested policies to manage weight loss in combat sports include: i) shortening the time between the weigh-in and competition [64]; ii) introducing more weight classes so that there are smaller gaps between weight classes [23]; iii) not allowing athletes multiple attempts to weigh-in, which may make the process more difficult for athletes losing larger amounts of weight [64]; iv) checking weight at an allocated point before the bout and then only allowing athletes to lose a certain amount of weight from there [23,64]; v) the implementation of athlete education programs to encourage athletes and support staff to choose safer weight loss practices [23] and; vi) implementing hydration testing requirements for athletes to compete [64]. While certain suggestions such as an increased number of weight classes and the implementation of athlete education programs appear sound, other suggestions may have unintended consequences. Indeed, shortening the time between the weigh-in and competition could result in impaired recovery from the rapid weight loss and increase the risk of brain injury during competition [23,56]. Further, the implementation of a weigh-in weeks out from the competition to decide upon a weight loss limit may encourage athletes to cut weight for the newly introduced weigh-in, thus increasing the frequency of weight-cutting. Finally, there are currently many controversies and limitations when it comes to testing hydration in humans and there are no tests that are accurate enough to give reliable and valid results, especially in the case of acute dehydration and rehydration [9,48]. It is also important to note that many athletes consider the weight-cutting process an essential part of the preparation for a competition, as it gives them a sense of control [63]. Additionally, the process of hosting athlete weigh-ins is a part of the entertainment and promotion of combat sports competitions. These factors outline potential motivations for both athletes and combat sport event organisers to attempt to maintain the current practices of weight-cutting. As a result, a careful conversation on how to best manage weight-cutting in combat sports will be required in the future. Such a conversation should be carefully considered and evidence-based, as well as involving scientists, athletes, coaches, government officials and private event organisers. However, for such a conversation to be effective, substantially more research is required to inform opinion and interpretation in this field, in particular investigating the physiological and psychological influence of weight-cutting, as well as its sociology within combat sports cultures. Conducting such research will allow for well-informed best-practice guidelines to be developed.

## 8. Conclusions and Practical Applications

Weight-cutting is commonplace in many, if not all, combat sports despite the potential negative affect on health and performance. The methods of weight loss range from gradual dieting to severe thermal exposure to induce significant water loss. Regarding physical performance, it appears clear that more severe weight-cuts (~5% body mass in under 24 h) will impair repeat-effort capacities, but it is unclear if the benefits from fighting in a lighter weight class outweigh such negative effects, which future research should look to investigate. In particular, research should investigate the influence of weight cutting on performance following longer (~24 h) recovery times. Weight-cutting may result in many negative health effects, and of particular concern is the potential for increased risk of brain injury which should be a topic of future research. There are several potential strategies for minimising the prevalence and magnitude of weight-cutting practices, which may be of importance in the future. However, there is a paucity of data on their effectiveness, which should be investigated by future research. There are also strategies that can be utilised during the recovery period which can maximise athlete health and performance. These strategies include aggressively replenishing glycogen, body water and electrolytes, while carefully considering athlete gastric comfort. It is unlikely that weight-cutting in combat sports will be stopped anytime soon, however a combination of strategies to minimise weight-cutting and to improve recovery during recovery periods may drastically improve the culture around weight-cutting and help athletes maximise their health and competitive performance.
